# Forecast and Analysis on Reducing China’s CO_2_ Emissions from Lime Industrial Process

**DOI:** 10.3390/ijerph16030500

**Published:** 2019-02-11

**Authors:** Qing Tong, Sheng Zhou, Yuefeng Guo, Yang Zhang, Xinyang Wei

**Affiliations:** 1 Institute of Energy, Environment, and Economy, Tsinghua University, Beijing 100084, China; tongqing@tsinghua.org.cn (Q.T.); zhshinet@tsinghua.edu.cn (S.Z.); 2 State Key Laboratory of Virtual Reality Technology and Systems & Suzhou Institute of Beihang University, Suzhou 215200, China; guoyuefeng1978@163.com (Y.G.); stevenyang030@163.com (Y.Z.); 3 Department of Accounting and Finance, School of Business, Macau University of Science and Technology, Macau, China

**Keywords:** forecast, CO_2_ emission reductions, lime industrial process, China, scenario analysis

## Abstract

China greenhouse gas inventories show that CO_2_ emissions from the lime industrial process are large scales and closely related to the development of its downstream industries. Therefore, there is high importance to analyze and forecast on reducing China’s CO2 emissions from lime industrial process. The aims of this paper are to make up the research gaps in China and provide a quantitative reference for related authorities to formulate relevant policies. The prediction method in this paper is consistent with the published national inventory, which is an activity data based method to predict carbon dioxide emissions from the industrial process of four categories of lime products. Three future scenarios are assumed. The business as usual scenario (BAU) is a frozen scenario. There are two emission reduction scenarios (ERS and SRS) assumed under different emission reduction strength considering combined industrial process CO_2_ emission reduction approaches from both the production side and the consumption side. The results show that between 2020 and 2050, China’s lime industrial process has an increasingly significant CO_2_ emission reduction potential, enabling both emission intensity reductions and total emission reductions to be achieved simultaneously. Based on the simulation results from emission reduction scenarios, compared with 2012 level, in 2050, the emission intensity can be reduced by 13–27%, the total lime production can be reduced by 49–78%, and the CO_2_ emissions in the lime industrial process can be reduced by 57–85%.

## 1. Introduction

Industrial process emissions refer to greenhouse gas emissions caused by industrial activities (chemical or physical material transformation) not related to energy during the industrial processes [1]. Specifically, emissions from industrial processes accounted for about 17% of the total CO_2_ emissions from China’s energy-intensive manufacturing industries [2].

In China, the raw material for lime production is mainly limestone, which is produced in lime calcination kilns. In the calcination production process, the main chemical composition of limestone, the calcium carbonate (CaCO_3_) is thermally decomposed into calcium oxide and carbon dioxide. Calcium oxide is the effective chemical component of lime, commonly referred to as quicklime. From the perspective of the lime industry, industrial process emissions are the primary source of carbon dioxide emissions, accounting for nearly 70% of the whole industry’s emissions [3], far higher than the proportion of emissions from energy activities, and of the pollution produced by the automobile traffic [4,5,6,7].

**Figure 1:**
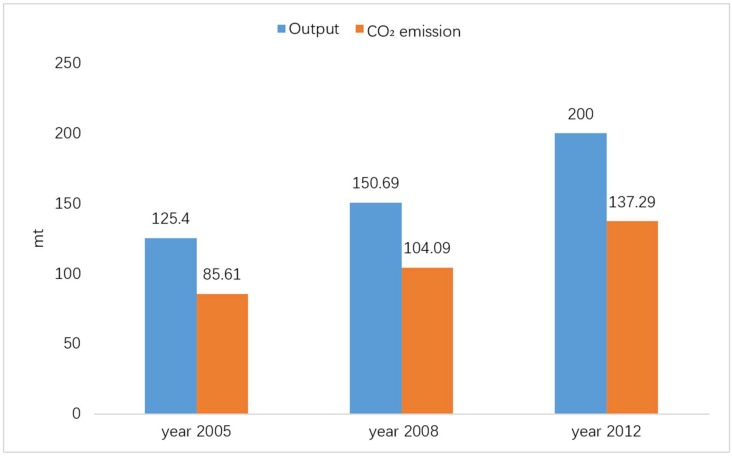
Greenhouse gas inventory data from China’s lime industrial process.

The Chinese government released China’s greenhouse gas inventories in 1994 [7], 2005 [8,9], 2008 [10] and 2012 [11]. The 1994 national greenhouse gas inventory was applied in the early period and it cannot provide too much reference for current research. The subsequent three inventories have consistency in the accounting methodology and data caliber of the lime industrial process. They combined the methodologies of the Revised 1996 IPCC (Intergovernmental Panel on Climate Change) Guidelines for National Greenhouse Gas Inventories (1996 IPCC Guidelines), the Good Practice Guidance and Uncertainty Management in National Greenhouse Gas Inventories (GPG2000) [12], and the 2006 IPCC Guidelines for National Greenhouse Gas Inventories [13] with China’s actual situation. Based on the differentiation in emission factors and uses, lime products are divided into four categories, namely construction lime, metallurgical lime, chemical lime and other lime. The emissions are calculated in a bottom-up manner. The greenhouse gas inventory of the lime industrial process in Figure 1 shows that as the capacity of the lime industry expands, the carbon dioxide emissions of its industrial process present an increasing trend. From 2005 to 2008, the annual average growth rate of China’s total lime production was 6.32%, and the annual growth rate of lime industrial emissions was 6.73%. From 2008 to 2012, the annual average growth rate of China’s total lime production was 7.33%, and the annual growth rate of lime industrial emissions was 7.17%. The Figure 1 shows greenhouse gas inventory data, produced in the China by the lime industrial process.

The emission factors of the lime industrial process are determined by the chemical composition of raw materials. At present, a normalized statistical mechanism for the emission factors of the lime industrial process has not been established yet in China. Also, there is no CO_2_ emission reduction technology that has been commercialized and specifically designed for the lime industrial process. Therefore, the three published Chinese greenhouse gas inventories use the same emission factors which are classified by different uses of lime products. The source of these emission factors is a survey of typical lime enterprises jointly conducted by Tsinghua University and the China Lime Association. Table 1 shows the lime industrial process emission factors (t CO_2_/t lime) in China inventory.

Due to the existence of a large number of small and medium-sized enterprises and township enterprises, existing literature looking into the lime industry emission mitigation is relatively sparse not only in China but also on a global scale. On international level, the three versions of IPCC Guidelines [1,12,13] are merely methodological tools for national greenhouse gas inventory without guidance on certain sectors’ emission mitigation. The IPCC Fifth Assessment Report (AR5) [14] discusses industrial mitigation of climate change with the focus on such key industries as cement, iron & steel manufactures, where lime industry is not included. There is no other international literature forecasting on the lime industrial process emission mitigation. On national level, necessary statistical and forecast data cannot be obtained in China. The official greenhouse gas emission data for lime production released by the Chinese government is only provided in the four years mentioned above. Research on greenhouse gas emission and emission reduction in China’s lime industry is limited, mainly at the level of current situation analysis and emission inventories, and there is no published literature on greenhouse gas emission forecast for the lime industrial process. In terms of existing literature on current status analysis and emissions inventories, their limitations are stated below:

1. Emission factors: International emission factors are used because of data availability and other reasons. Due to the geographical differences in limestone raw materials, the international emission factors for lime industrial processes are mostly higher than China’s national and specific emission factors shown in Table 1. Therefore, the default value of global average emission factor given by IPCC is 0.75 tCO_2_/t lime [12,13]. Based on this estimate, using the IPCC default emission factor will result in an over-estimation of 7–9% of emissions. In addition, some Chinese literature adopts the EU’s emission factor of 0.785 tCO_2_/t lime [15,16], and the over-estimation will reach 11–13% of emissions.
2. Activity data: Some studies have not classified lime products [3,15,16,17,18,19], which cannot reflect the impact of changes in product structure on greenhouse gas emissions during lime production. Some literature also focuses on the study of metallurgical lime, a single product with better data availability [20,21]. However, based on the 2012 inventory data, both the metallurgical lime production and emission account for less than 50% of the total lime.
3. Emission reduction approaches: Most of the existing research focuses on energy-saving technologies on the lime production side [22,23,24,25]. There is also a small amount of literature conducting a prospective study on carbon dioxide capture and reuse technologies which have not yet been commercialized in the lime industry [16,26,27,28]. However, there is currently no published literature in China which considers emission reduction policies and technologies from both the lime production and demand sides, and provides quantitative research results.

China greenhouse gas inventories show that CO_2_ emissions from the lime industrial process are large scales and closely related to the development of its downstream energy-intensive industries, such as steel and calcium carbide. Therefore, there is high importance to analyze and forecast on reducing China’s CO2 emissions from lime industrial process. The aims of this paper are to make up the above research gaps in China and provide a quantitative reference for related authorities to formulate relevant policies as follows:

1. Emission factors: In terms of caliber, the forecast method of this paper is consistent with the published national inventories. Emission factors are classified by 4 different uses of lime products to improve accuracy and consistency of data series.
2. Activity data: In consistence with emission factors’ caliber, activity data are also classified into the same 4 categories by use. Different lime categories are analyzed respectively in depth to improve completeness and accuracy of data series.
3. Emission reduction approaches: Starting from the lime production and demand sides simultaneously, this paper combines with the scenario analysis method in order to measure the comprehensive emission reduction effects of various emission reduction pathways. It provides China with CO_2_ emissions forecasting and emission reduction path analysis for its lime industrial process by 2050.

The remainder of this paper is organized as follows. In Section 2, we detail our research method and scenario assumptions. In Section 3, we discuss the results, including emission factors, Activity data, CO_2_ emissions from the lime industrial process as well as implied emission factors. In Section 4, we draw the conclusions and provide relevant policy implications for related administrative departments and policymakers.

## 2. Methodology and Scenario Assumptions

### 2.1. Methodology

#### 2.1.1. Forecasting Carbon Dioxide Emissions

The prediction method is consistent with the published national inventory, which is an activity data based method to predict carbon dioxide emissions from the industrial process of four categories of lime products, as shown in formula (1). Based on the latest available data in 2012, the activity data (lime outputs) and emission factors for the target year are respectively predicted. Multiplying the activity data by its corresponding emission factor is the carbon dioxide emission from the industrial process of a certain lime category in the target year. The carbon dioxide emissions from the industrial process of four categories of lime are added up to obtain the total carbon dioxide emissions from the industrial process of the lime industry in the target year.

(1) $$E_{\mathrm{CO}2}=\underset{i}{\sum }({\mathrm{EF}}_{i}\times {\mathrm{AD}}_{i})$$

where:

$E_{\mathrm{CO}2}$ is total carbon dioxide emissions from lime industrial process in the target year (mtCO_2_);
i expresses the category of lime products, including four categories: construction lime, metallurgical lime, chemical lime and other lime;
EF_i_ is the carbon dioxide emission factor of the industrial process of lime category i in the target year (tCO_2_/t);
AD_i_ is activity data of the target year, that is, the output of lime category i (mt).

#### 2.1.2. Emission Factor Prediction

For the prediction of emission factors, the application of lime production-based emission reduction technology and its influence are mainly considered. Lime production is a traditional industry. As far as the lime industrial process itself is concerned, what happens is a simple chemical reaction. The emission factor is only related to the chemical composition of raw materials, and has nothing to do with the improvement of production efficiency and energy efficiency. As a result, China has no commercially available carbon-dioxide emission reduction technology specifically for the lime industrial process. However, according to the literature study review, a technology named CALGERGY, currently at the research stage, can absorb 90% carbon dioxide emissions in the lime industrial process by planting microalgae within the boundaries of lime enterprises [26]. The algae that absorb carbon dioxide are then used to produce biodiesel to substitute fossil fuels. There are some other carbon capture technologies applicable to the lime industry under research and development which would make emission reduction effects by reusing the captured carbon dioxide in iron and steel, chemical engineering and food industries. [27,28,29] The efficiency of all the above carbon capture technologies for the lime industry is 90%. [26,27,28,29] Carbon dioxide emission factors in the lime industrial process can be quantitatively predicted by formula (2).

(2) $${\mathrm{EF}}_{i}={\mathrm{EF}}_{i0}\times \left(1-T_{i}\right)+{\mathrm{EF}}_{i0}\times \left(1-90\%\right)\times T_{i}$$

where:

i expresses the type of lime, including four categories of lime: construction lime, metallurgical lime, chemical lime and other lime;
EF_i_ is the carbon dioxide emission factor of the industrial process of category i lime in the target year (tCO_2_/t);
${\mathrm{EF}}_{i0}$ is the carbon dioxide emission factor of the industrial process of category i lime without emission reduction technology (tCO_2_/t);
$T_{i}$ is technology penetration rate of the CCU.

#### 2.1.3. Forecast on Activity Data

The prediction of activity data (outputs) needs to start from the demand side of lime for different uses. The specific prediction methods of different categories of lime by usages are as follows:

1. Metallurgical lime: the flow direction of products is basically as the raw material of long-process steelmaking industry, which has a mass balance relationship [20] with crude steel output, and can be expressed as formula (3). With the increase of the proportion of short-process electric arc furnace steelmaking technology and the progress of long-process steelmaking technology in the future, the metallurgical lime consumption coefficient has a certain space to decrease [21].
  (3) $${\mathrm{AD}}_{\mathrm{sy}}=S_{y}\times C_{sy}$$
  where:
  ${\mathrm{AD}}_{\mathrm{sy}}$ is metallurgical lime output in the target year (mt);
  $S_{y}$ is crude steel output in the target year (mt);
  $C_{sy}$ is metallurgical lime consumption coefficient in the target year (t lime/t crude steel).
2. Chemical lime: the flow direction of products is mainly as the raw material of calcium carbide, and there is a mass balance relationship with the calcium carbide output [29], which can be expressed as formula (4).With the increase of technology proportion of hermetic calcium carbide furnace with higher energy efficiency and lower raw material consumption [30], as well as the development of alternative raw material technology [31] in calcium carbide industry, the consumption coefficient of lime in the chemical industry has certain space to decrease.
  (4) $${\mathrm{AD}}_{\mathrm{py}}=P_{y}\times C_{py}$$
  where:
  ${\mathrm{AD}}_{\mathrm{py}}$ is chemical lime output in the target year (mt);
  $P_{y}$ is calcium carbide output in the target year (mt);
  $C_{py}$ is chemical lime consumption coefficient in the target year (t lime/t calcium carbide).
3. Construction lime and other lime: the products of lime are directly used as construction materials [32], disinfectant [33,34] and flue gas desulfurization [35], etc. Most of these two kinds of lime products are produced by township enterprises which of these below the scale, with backward production capacity and serious pollution. In the future, with the in-depth implementation of policies to eliminate backward production capacity [36,37], and the use of biomass [38] or carbide sludge [39,40] to produce new environmental-friendly materials to replace the use of construction lime and other lime, the consumption and output of these two types of lime can be significantly reduced. In the prediction study, the decrease of the two types of lime output in the target year relative to the base year (2012) will be assumed, so as to calculate the output of construction lime and other lime by using formula (5).
  (5) $${\mathrm{AD}}_{\mathrm{oy}}={\mathrm{AD}}_{y0}\times \left(1-D_{oy}\right)$$
  where:
  ${\mathrm{AD}}_{\mathrm{oy}}$ is total construction lime and other lime output in the target year (mt);
  ${\mathrm{AD}}_{y0}$ is total construction lime and other lime output in the base year (mt);
  $D_{oy}$ is the decrease percentage of construction and other lime output in the target year relative to the base year.

#### 2.1.4. Forecast on Implied Emission Factors

Based on the prediction of total carbon dioxide emissions from the lime industrial process, the Implied Emission Factors (IEF) of the lime industrial process can be further calculated according to formula (6). The change of IEF is related to the progress of lime production technology and the optimization of the product structure.

(6) $$\mathrm{IEF}=E_{\mathrm{CO}2}/\mathrm{AD}$$

where:

IEF is implied emission factors of the lime industrial process in the target year (tCO_2_/t);
$E_{\mathrm{CO}2}$ is total carbon dioxide emissions from lime industrial process in the target year (mtCO_2_);
AD is total lime outputs in the target year (mt).

### 2.2. Scenario Assumptions

Three scenarios are assumed for predictive analysis. The business as usual scenario (BAU) is a frozen scenario in which the emission factors are consistent with the base year and the total outputs of lime are consistent with the projected peaking value during 2020–2050. In other words, it assumed in the BAU scenario that there would be a plateau after lime output peaking. There are two emission reduction scenarios assumed under different emission reduction strength. Both of the emission reduction scenarios, the ERS and SRS, consider combined industrial process CO_2_ emission reduction approaches from both the production side and the consumption side. Production-based approaches include a series of CCU technologies with different technology penetration rate assumption in two emission reduction scenarios. Consumption-based approaches include control on crude steel outputs, reduction of metallurgical lime consumption coefficient, control on calcium carbide outputs, reduction of chemical lime consumption coefficient and reduction of the output of construction lime and other lime by substituting materials. There is no increasing emission trend scenario assumed in this paper since the variation of CO_2_ emissions from lime industry are related to technology progress and demand of its downstream industries. Based on the scenario parameters extracted from existing literatures, China will basically achieve industrialization by 2020, there is potential of technology progress in lime industry and its downstream demand will peak around 2020. Therefore, there would be little possibility of an increasing CO_2_ emission trend in lime industry.

Detailed scenario assumptions are as follows:

#### 2.2.1. Business as Usual (BAU)

The business as usual scenario (BAU) is a frozen scenario. Regardless of technological progress, the emission factors of each target year are consistent with the base year, as shown in Table 1. According to the 13th Five-year Plan for the Development of the Lime Industry [37], the total output of lime in 2020 is expected to be 250 million tons. Thereafter, total lime production in each target year is frozen at 2020 levels. The product structure of each target year is consistent with the base year 2012, and the activity data of four lime products are predicted accordingly, as shown in Table 2.

#### 2.2.2. Emissions Reduction Scenario (ERS)

Emissions reduction scenario (ERS) considering carbon dioxide emission reduction approaches of industrial processes in both the production-based and the consumption-based of lime, as follows:

Production-based approaches: To promote the CCU technologies, the technology penetration rate will reach 15% by 2050.

Consumption-based approaches:

1. Control crude steel outputs. Crude steel production prediction data under the BAU scenario of *Reinventing Fire: China* [41] (ERI, September 2016) was quoted. This research is in accordance with the current data of China’s main industrial product output, and the predicted results are in line with the development planning and trend of related industries.
2. Reduce metallurgical lime consumption coefficient. The metallurgical lime consumption coefficient calculated according to the emission inventory in 2012 is 0.130t/t steel. Taking this as the benchmark, the metallurgical lime consumption coefficient decreases by 0.001t/t steel each year [21] and it will decrease to 0.092t /t steel by 2050.
3. Control calcium carbide outputs. The prediction data of calcium carbide output in the BAU scenario of *Reinventing Fire: China* (ERI, September 2016) was quoted.
4. Reduce chemical lime consumption coefficient. Assuming that all advanced technologies of closed electric arc furnace are adopted in 2050, the consumption coefficient of lime in the chemical industry will decrease linearly from 1.284t/t calcium carbide in 2012 to 0.909t/t calcium carbide in 2050 [31].
5. Reducing the output of construction lime and other lime by substituting materials. It is assumed that construction lime and other lime output will drop to 50% of 2012 production by 2050.

The corresponding target parameters of emission reduction approaches in this scenario are shown in Table 3.

#### 2.2.3. Strengthened Reduction Scenario (SRS)

The strengthened emission reduction scenario (SRS) and the emission reduction scenario (ERS) considering the same emission reduction approaches, but with greater intensity, as follows:

Production-based approaches: To promote the CCU technologies, the technology penetration rate will reach 30% by 2050.

Consumption-based approaches:

1. Control crude steel outputs. The prediction data of crude steel output in the remolding energy scenario of Reinventing Fire: China [41] (ERI, September 2016) was quoted.
2. Reduce metallurgical lime consumption coefficient. The metallurgical lime consumption coefficient calculated according to the emission inventory in 2012 is 0.130t/t steel. Taking this as the benchmark, the metallurgical lime consumption coefficient decreases by 0.0015t/t steel each year and it will decrease to 0.073t /t steel by 2050.
3. Control calcium carbide outputs. The prediction data of calcium carbide output in the remolding energy scenario of Reinventing Fire: China (ERI, September 2016) was quoted.
4. Reduce chemical lime consumption coefficient. Assuming that all advanced technologies of closed electric arc furnace are adopted in 2050 and some lime inputs are replaced by alternative raw materials, the consumption coefficient of lime in the chemical industry will decrease linearly from 1.284t/t calcium carbide in 2012 to 0.871t /t calcium carbide in 2050 [31].
5. Reducing the outputs of construction lime and other lime by substituting materials. It is assumed that construction lime and other lime output will drop to 10% of 2012 production by 2050.

The target parameters corresponding to emission reduction approaches in this scenario are shown in Table 4.

## 3. Result Analysis

### 3.1. Emission Factors

**Figure 2:**
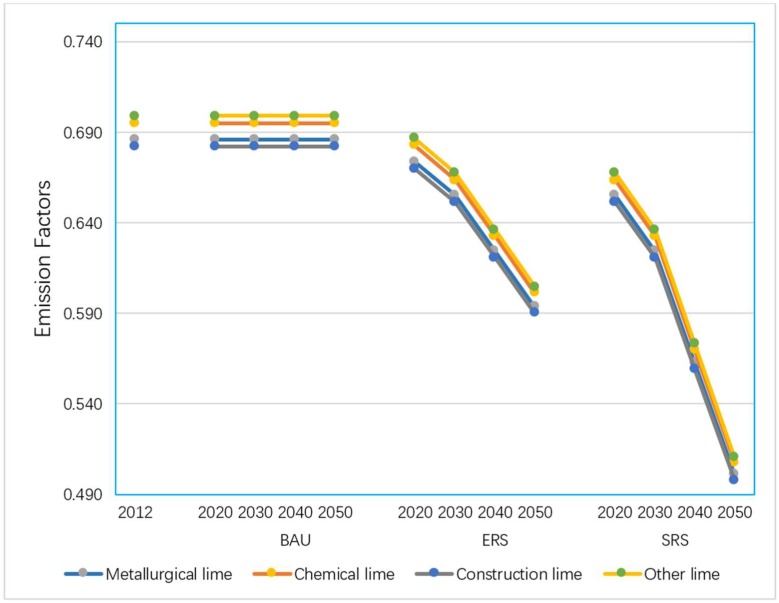
Emission factors for each scenario (t/t).

Since the penetration rate of emission reduction technology from limestone manufacture side in each scenario is not differentiated by lime product categories, the emission factor’s decline trend of each product is parallel to each other in each emission reduction scenario, as shown in Figure 2. By 2030, the emission factors for the two emission reduction scenarios will be 4% and 9% lower than their 2012 levels respectively. By 2050, the emission factors for the two emission reduction scenarios will be 13% and 27% lower than their 2012 levels respectively. The Figure 2 shows the emission factors for each scenario (t/t).

### 3.2. Activity Data

**Figure 3:**
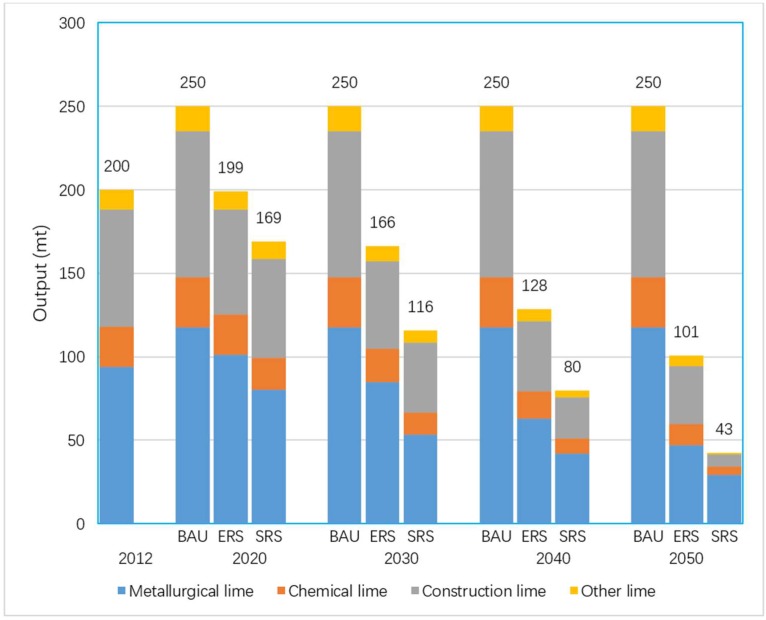
Lime productions in each scenario.

According to the China Industrialization Process Report released by the Chinese Academy of Social Sciences, China has entered the late stage of industrialization and will basically achieve industrialization by 2020 [42]. The forecast results in this paper also confirm with this prediction. The lime productions in the BAU scenario will peak in 2020. According to industry planning targets, the peak value is set to 250 mt. For both emission reduction scenarios, lime productions will peak before 2020. By 2030, lime productions in the two emission reduction scenarios will fall to 166 and 116 mt, respectively. Relative to the 2012 level, the decline is 17% and 42% respectively. By 2050, lime productions of the two emission reduction scenarios will further drop to 101 and 43 mt. The decline is 49% and 78%, respectively, relative to the 2012 level. The details are shown in Figure 3.

**Figure 4:**
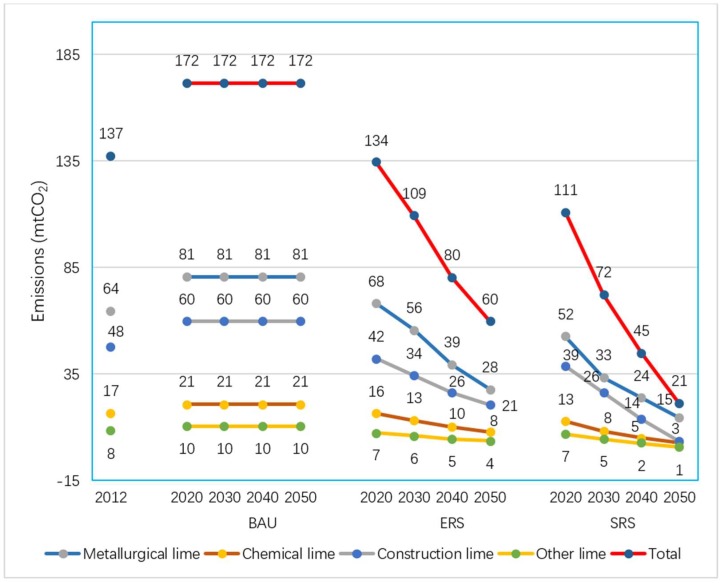
CO_2_ emissions from lime production in each scenario.

The production forecast shows that the product structure of the ERS scenario will not change much. The proportion of metallurgical lime, which occupies the highest proportion, varies from 47% to 51% in each target year, followed by the proportion of construction lime fluctuating between the range of 32–35%. The product structure of the SRS scenario will change significantly from 2030, and the proportion of metallurgical lime rise to 69%, while the proportion of construction lime falls to 16% by 2050. The Figure 3 shows for each scenario, the lime productions. The Figure 4 shows for each scenario, the CO_2_ emissions from lime production.

### 3.3. CO_2_ Emissions from Lime Industrial Process

In correspondence with the three scenario assumptions, there would be three trends of future lime industrial process emissions. The lime industrial process emissions from the BAU scenario will reach a peak of 172 mt CO_2_ in 2020 and retain a plateau during the whole forecasting period. For both emission reduction scenarios, process emissions will peak before 2020 and decrease continuously afterwards. The main difference of the emission trends between the two emission reduction scenarios are the decrease ranges due to different emission reduction strength assumptions. By 2030, the lime industrial process emissions of the two emission reduction scenarios will drop to 109 and 72 mt CO_2_, respectively. The decline is 20.4% and 47.4%, respectively, relative to the 2012 level. By 2050, lime production emissions in the two reduction scenarios will further decline to 60 and 21 mt CO_2_. The decline is 56.2% and 84.7%, respectively, relative to the 2012 level. Details are shown in Figure 4. Due to the superposition of the emission reduction effects from lime’s manufacture side and demand side, the decline of CO_2_ emissions from the lime industrial process is faster than the decline in lime outputs.

### 3.4. Implied Emission Factors

**Figure 5:**
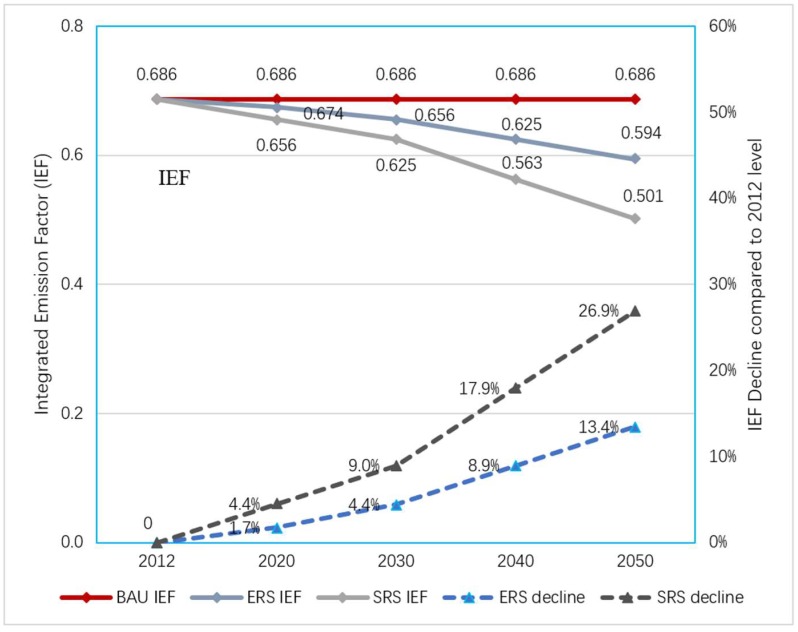
Implied emission factor and decline trend for each scenario.

The implied emission factor for the BAU scenario is constant at 0.686 tCO_2_/t lime. By 2030, the implied emission factors for the two emission reduction scenarios will fall to 0.656 and 0.625 tCO_2_/t lime, which are 4.4% and 9.0% lower than the 2012 levels, respectively. By 2050, the implied emission factors for the two emission reduction scenarios will further decrease to 0.594 and 0.501 tCO_2_/t lime, which are 13.4% and 26.9% lower than the 2012 levels, respectively. Details are shown in Figure 5.

### 3.5. Emission Reduction Effects

The emission reductions and emission reduction rates achieved in each target year of different scenarios are used to measure their emission reduction effects. The emission reduction effect is the concept of relative quantity and is related to the setting of the baseline. The following two types of benchmarks are used for measurement.

**Figure 6:**
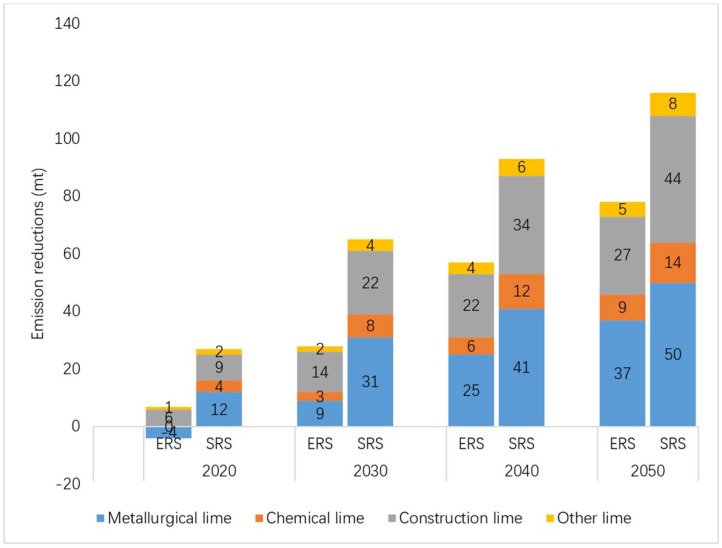
Emission reductions for different lime products in two emission reduction scenarios based on 2012 emission levels.

Based on the actual emissions in 2012, by 2030, the emission reductions of the two emission reduction scenarios are 28 and 65 mt CO_2_, with emission reduction rates of 20.5% and 47.3%, respectively. By 2050, the emission reductions of the two emission reduction scenarios will reach 78 and 116 mt CO_2_, with emission reduction rates of 56.5% and 84.5%, respectively. Results are shown in Figure 6.

**Figure 7:**
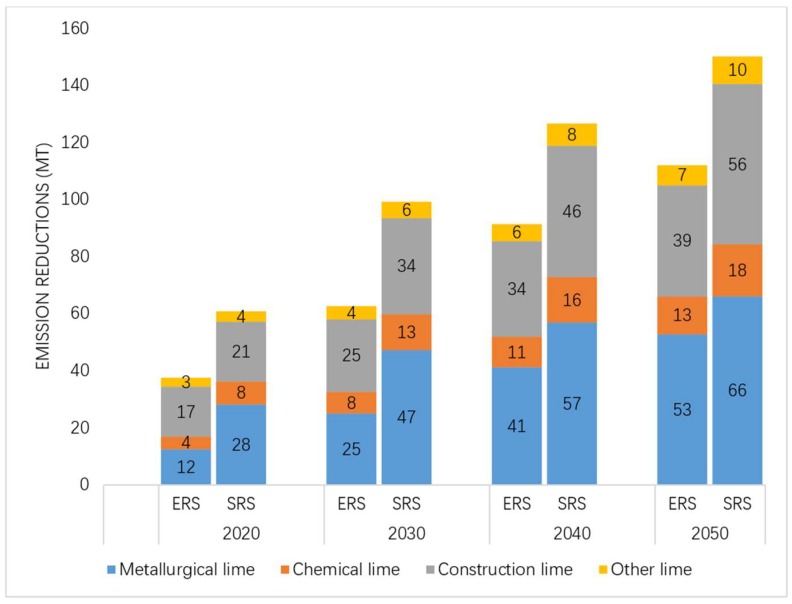
Emission reductions for different lime products in two emission reduction scenarios based on BAU emission levels.

**Figure 8:**
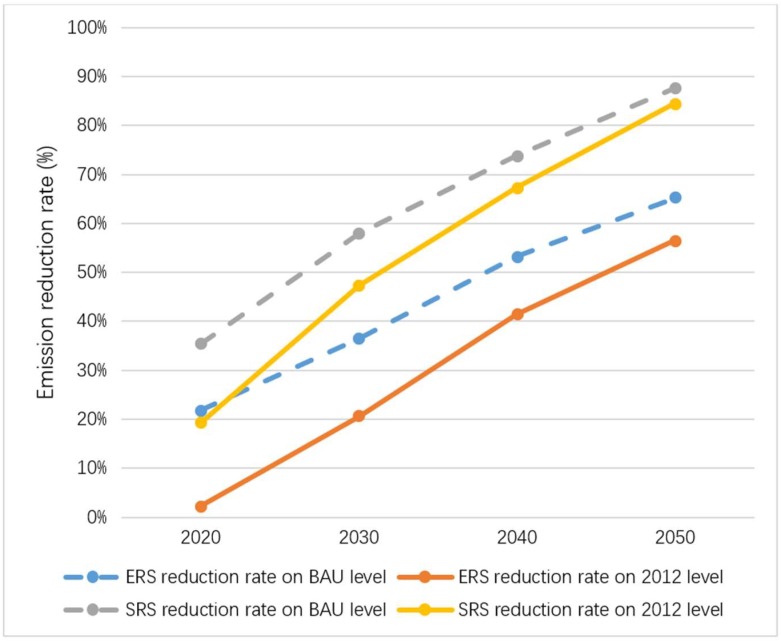
Emission reduction rates of different lime products in two emission reduction scenarios.

Based on the emission forecast data for the same target year in the BAU scenario, by 2030, the emission reductions for the two emission reduction scenarios are 63 and 99 mt CO_2_, with reduction rates of 36.4% and 57.9%, respectively. By 2050, the emission reductions of the two emission reduction scenarios will reach 112 and 151 mt CO_2_, with emission reduction rates of 65.2% and 87.6%, respectively. Results are shown in Figure 7. Emission reduction rates of different lime products in two emission reduction scenarios are shown in Figure 8.

Since metallurgical lime and construction lime account for the highest proportions of both productions and CO_2_ emissions in each scenario, they are also the two largest contributors to emission reductions. Among the emission reduction sources of the two emission reduction scenarios calculated using different baselines, the total proportion of metallurgical lime and construction lime in both 2030 and 2050 is over 80%. Figure 6 shows the emission reductions for different lime products in two emission reduction scenarios based on 2012 emission levels. Figure 7 shows the Emission reductions for different lime products in two emission reduction scenarios based on BAU emission levels. Figure 8 shows the emission reduction rates of different lime products in two emission reduction scenarios.

## 4. Conclusions

The traditional view is that the carbon dioxide emission reduction potential of the lime industrial process is small since there are limitations of existing literature on the industry’s current emissions analysis and even sparse literature looking into the lime industry emission mitigation not only in China but also on a global scale. However, this paper makes up the research gaps in China and provides a quantitative reference for related authorities to formulate relevant policies which considers the development of policies and technologies on lime production and demand sides in a comprehensive manner and sorts out various approaches of emission reductions. By combining with scenario analysis methods, this paper quantitatively estimates the pathways of comprehensive emission reduction approaches. Three future scenarios are assumed. The business as usual scenario (BAU) is a frozen scenario in which the emission factors are consistent with the base year and there would be a plateau after lime output peaking. There are two emission reduction scenarios (ERS and SRS) assumed under different emission reduction strength considering combined industrial process CO_2_ emission reduction approaches from both the production side and the consumption side. The results show that between 2020 and 2050, China’s lime industrial process has an increasingly significant CO_2_ emission reduction potential, enabling both emission intensity reductions and total emission reductions to be achieved simultaneously.

Emission intensities are the emission factors of various lime products and the implied emission factor of the whole industry. Under the preconditions of scenario analysis, the decrease of emission intensity would be achieved at different rate based on the technological progress and environmental protection policies on the lime production side. The lime production side emission reduction technology with future application prospects consists of a series of Carbon Capture and Utilization (CCU) technologies. The CCU technology penetration rate predicted in the two emission reduction scenarios reaches 5–10% in 2030 and 15–30% in 2050. On the hypothetical basis of this, the forecasted emission intensity is reduced by 4–9% in 2030 and 13–27% in 2050. Although CCU technology has obvious CO_2_ emission reduction effects, the high cost of abatement is the main constraint of technology promotion. The cost of abatement of CCU technology is expected to be 58–68 US $/tCO_2_ [43,44]. Now, the Chinese government has officially initiated the construction of a national carbon market [45], and research on promoting the development of low-carbon technologies in the high-carbon sectors by imposing carbon taxes is also being carried out simultaneously [46]. From the experience of the European Union and other foreign countries, the lime industry, which is a major greenhouse gas emitter, will be strictly constrained by the carbon emission control policy [47]. The internalization of environmental costs can overcome the constraints of CCU technology costs and accelerate technology promotion.

In addition to relying on technological progress on the production side to reduce emission intensity, it is more important to rely on technological progress and total volume control on the demand side in order to reduce the total amount of CO_2_ emissions in the lime industrial process. The main demand side of lime products, including steel, chemical and construction industries, all belong to the industries which are strictly controlled by the Chinese government in terms of production technology, production capacity and environmental protection [48]. With the opportunity for China to complete industrialization as well as the in-depth elimination of outdated industrial capacity and overcapacity in major industries, the output of major products will reach the peak before 2020. Technological progress and the R&D and application of alternative raw materials will continue to reduce the lime consumption factor on the demand side. Through the superimposing effect of policy and technology from the demand side, the lime production of the two emission reduction scenarios is 17% and 42% lower than the 2012 level respectively by 2030; the lime production of the two emission reduction scenarios will fall by 49% and 78% respectively by 2050 compared to 2012 levels. In addition, the strengthened emission reduction scenario also reflects the optimization of the lime product structure. Compared with the 2012 level, the proportion of high-quality metallurgical lime production increases by 12 percentage points in 2050. Moreover, the proportion of construction lime produced mainly by township enterprises decreases by 16 percentage points.

The forecast results of this paper show that with the implementation of the bilateral guidance of lime production and demand sides, by 2030, China can achieve 20.5–47.3% reductions based on the actual emissions in 2012. By 2050, China can reduce emissions by 56.5~84.5%. Based on the emission forecast data for the same target year in the BAU scenario, by 2030, emissions reductions of 36.4–57.9% can be achieved. By 2050, China can reduce emissions by 65.2–87.6%. Among the emission reduction sources of the two emission reduction scenarios calculated using different baselines, the total proportion of metallurgical lime and construction lime in both 2030 and 2050 is over 80%. Therefore, metallurgical lime and construction lime are key sources of CO_2_ emission reduction in China’s lime industrial process.

To achieve the above predicted emission reduction potential at lowest uncertainty, it is necessary to rely on both international and domestic policies. Main supportive international policies include the links of international carbon markets and stricter environmental indicators on import which would lead to decrease of lime industry’s downstream demand, the steel and chemical products. Chinese government would play an important role on its current industrial and environmental protection policies’ continuity and enhancement. It is crucial to implement national carbon market, carbon tax and shut down backward production facilities to promote technology progress and total output control and thus guarantee both emission intensity reductions and total emission reductions in predicted pathways.

## Figures and Tables

**Table 1:** Lime industrial process emission factors (t CO_2_/t lime) in China inventory.

Product Use	Metallurgical Lime	Chemical Lime	Construction Lime	Other Lime
Emission factor	0.686	0.695	0.682	0.699

**Table 2:** Lime production under the BAU scenario (mt).

Product Categories	2012	2020	2030	2040	2050
Metallurgical lime	94	118	118	118	118
Chemical lime	24	30	30	30	30
Construction lime	70	88	88	88	88
Other lime	12	15	15	15	15
Total	200	250	250	250	250

**Table 3:** Emission reduction approaches and target parameters under the ERS.

Classification	Emission Reduction Approaches/Target Parameters	Units	2020	2030	2040	2050
Production-based	Technology penetration rate of CCU	%	2%	5%	10%	15%
Consumption-based	Capacity cut target on crude steel	mt	830	760	620	510
	Metallurgical lime consumption coefficient	t/t	0.122	0.112	0.102	0.092
	Capacity cut target on calcium carbide	mt	20	18	16	14
	Chemical lime consumption coefficient	t/t	1.205	1.106	1.008	0.909
	Construction lime production decrease relative to 2012	%	10%	25%	40%	50%
	Other lime production decrease relative to 2012	%	10%	25%	40%	50%

**Table 4:** Emission reduction approaches and target parameters under the SRS.

Classification	Emission Reduction Approaches/Target Parameters	Units	2020	2030	2040	2050
Production-based	Technology penetration rate of CCU	%	5%	10%	20%	30%
Consumption-based	Capacity cut target on crude steel	mt	680	520	480	400
	Metallurgical lime consumption coefficient	t/t	0.118	0.103	0.088	0.073
	Capacity cut target on calcium carbide	mt	16	12	9	6
	Chemical lime consumption coefficient	t/t	1.197	1.088	0.980	0.871
	Construction lime production decrease relative to 2012	%	15%	40%	65%	90%
	Other lime production decrease relative to 2012	%	15%	40%	65%	90%
